# Dynamic Regulation of Ero1α and Peroxiredoxin 4 Localization in the Secretory Pathway*[Fn FN2]

**DOI:** 10.1074/jbc.M113.467845

**Published:** 2013-08-26

**Authors:** Taichi Kakihana, Kazutaka Araki, Stefano Vavassori, Shun-ichiro Iemura, Margherita Cortini, Claudio Fagioli, Tohru Natsume, Roberto Sitia, Kazuhiro Nagata

**Affiliations:** From the ‡ Department of Molecular and Cellular Biology, Institute for Frontier Medical Sciences, Kyoto University, 53 Kawahara-cho, Shogoin, Sakyo-ku, Kyoto 606-8397, Japan,; the § Laboratory of Molecular and Cellular Biology, Faculty of Life Sciences, Kyoto Sangyo University, Motoyama, Kamigamo, Kita-ku, Kyoto 603-8555, Japan,; the ‖ Università Vita-Salute San Raffaele Scientific Institute, Division of Genetics and Cell Biology, Via Olgettina 58, I-20132 Milan, Italy,; the ¶ Molecular Profiling Research Center for Drug Discovery, National Institute of Advanced Industrial Science and Technology, 2-4-7 Aomi, Koto-ku, Tokyo 135-0064, Japan, and; the ** Medical Industry Translational Research Center, Fukushima Medical University, 1 Hikarigaoka, Fukushima 960-1295, Japan

**Keywords:** Endoplasmic Reticulum (ER), Oxidase, Peroxiredoxin, Thiol, Trafficking, Ero1, Prx4, Retention Mechanism

## Abstract

In the early secretory compartment (ESC), a network of chaperones and enzymes assists oxidative folding of nascent proteins. Ero1 flavoproteins oxidize protein disulfide isomerase (PDI), generating H_2_O_2_ as a byproduct. Peroxiredoxin 4 (Prx4) can utilize luminal H_2_O_2_ to oxidize PDI, thus favoring oxidative folding while limiting oxidative stress. Interestingly, neither ER oxidase contains known ER retention signal(s), raising the question of how cells prevent their secretion. Here we show that the two proteins share similar intracellular localization mechanisms. Their secretion is prevented by sequential interactions with PDI and ERp44, two resident proteins of the ESC-bearing KDEL-like motifs. PDI binds preferentially Ero1α, whereas ERp44 equally retains Ero1α and Prx4. The different binding properties of Ero1α and Prx4 increase the robustness of ER redox homeostasis.

## Teaser

**Background:** Ero1α and peroxiredoxin 4 contribute to disulfide formation in the early secretory compartment (ESC), but lack known retention signals.

**Results:** Retention and localization of Ero1α and peroxiredoxin 4 are maintained through multistep and pH-dependent interactions with PDI and ERp44 in ESC.

**Conclusion:** PDI and ERp44 dynamically localize Ero1α and peroxiredoxin 4 in ESC.

**Significance:** The levels and localization of four interactors allow differential ESC redox control.

## Introduction

Secretory or membrane proteins attain their native state in the ER,^5^ under the assistance of a vast array of resident chaperones and enzymes. Formation, cleavage, or rearrangement of disulfide bond is catalyzed by oxidoreductases of the protein disulfide isomerase (PDI) family, which in humans lists over 20 members (1). The C*XX*C motifs in thioredoxin-like active domains, so-called a-domains, mediate disulfide interchange reactions. Redox-inactive domains, or b-domains, of similar structure but lacking C*XX*C motifs are frequently found in PDI family members. In PDI, for instance, the two redox-active domains (a- and a′-domain) are separated by the b- and b′-domains (a-b-b′-a′). The b′-domain provides a hydrophobic pocket onto which client proteins and ER oxidoreductin-1 (Ero1) molecules dock (2, 3).

ERp44 has an a-b-b′ domain organization (4) and plays important roles in the early secretory compartment (ESC) (5). Unlike PDI and other KDEL-bearing proteins, ERp44 accumulates in the ER-Golgi intermediate compartment (ERGIC) and cis Golgi (6, 7). In its a-domain, ERp44 has a conserved redox motif, CRFS, whose cysteine (Cys-29) is used to form mixed disulfides with IgM, adiponectin, and other client proteins for thiol-dependent quality control (8–10). ERp44 binds and regulates Ero1α and β, two key ESC-resident oxidases (11), and displays pH-dependent conformational change in ESC to prominently retrieve Ero1 and premature secretory proteins from the ERGIC to the ER (12).

Upon transferring disulfide bonds to incoming client proteins, PDI can be efficiently reoxidized by members of the Ero1 family (Ero1α and Ero1β in mammals). As these flavoproteins use oxygen as an electron acceptor generating hydrogen peroxide as a byproduct, the question arose as to how professional secretory cells could fold abundant proteins rich in disulfide bonds with limiting oxidative stress. A solution of this paradox came with the discovery that peroxiredoxin 4 (Prx4) can promote *de novo* disulfide bond formation by utilizing hydrogen peroxide (13, 14). Furthermore, it has been recently revealed that mice with double knock-out of both oxidases exhibit lower birth rate and scurvy, whereas mice with single knock-out (Ero1 or Prx4) exhibit modest effect, indicating mutual complementarity between Ero1 and Prx4 (15). Surprisingly, however, neither Prx4 nor Ero1 contains known ER retention signals (supplemental Fig. 1). Ero1α interacts with PDI and ERp44 (16) and to a minor extent with other family members, including ERp57, ERp46, ERp18, P5, and ERp72 (17–19) In line with their preferential binding, ERp44 and PDI can efficiently retain overexpressed Ero1α (20). On the other hand, it has been unclear how Prx4 is retained in the ER (21).

In this study, we investigated the mechanisms that control the intracellular localization of Prx4. Our findings reveal that Prx4 shares a similar stepwise retention mechanism with Ero1α, in which ERp44 functions as a backup for PDI; when PDI is down-regulated, Ero1α and Prx4 are retained by ERp44 in the downstream compartment of the ER. Such dynamic regulation of two main ER oxidases seems important for maintaining redox homeostasis in the ESC because the expression of Ero1α and Prx4 endowed with KDEL motifs caused hyperoxidizing environment in the ER.

## EXPERIMENTAL PROCEDURES

### 

#### 

##### Cells and Antibodies

HeLa and HEK293 cells were cultured in Dulbecco's modified Eagle's medium with 10% fetal bovine serum and antibiotics. The primary antibodies used in the experiments were: mouse monoclonal anti-GFP (Roche Applied Science, Basel, Switzerland), mouse monoclonal anti-HA (Cell Signaling Technology), mouse monoclonal and rabbit polyclonal anti-FLAG (Sigma-Aldrich), mouse monoclonal anti-Prx4 (Abcam, Cambridge, UK), mouse monoclonal anti-Ero1α (Abcam for Western blot and Santa Cruz Biotechnology for immunofluorescence), mouse monoclonal anti-β-actin (Millipore), mouse monoclonal anti-ERGIC53 (Enzo Chemical Laboratories), rabbit polyclonal anti-ERp46 (Santa Cruz Biotechnology), chicken polyclonal anti-P5 (Santa Cruz Biotechnology), rabbit polyclonal anti-PDI (StressGen Biotechnologies Corp.), rabbit polyclonal anti-ERp44 (reported by Ronzoni *et al.* (22)), rabbit polyclonal anti-ERp72 (Santa Cruz Biotechnology), and rabbit polyclonal calnexin (Cell Signaling). The secondary antibodies used in the experiments were: HRP-anti-rabbit IgG, HRP-anti-mouse IgG, Alexa Fluor 488 anti-rabbit or -mouse, and Alexa Fluor 546 anti-rabbit or -mouse (Invitrogen).

##### Construction of Plasmids

Human Prx4, PDI (wild type or AA mutant), or Ero1α (wild type or C94A mutant) cDNA with a FLAG tag or with a FLAG tag and KDEL sequence at the C terminus was generated by PCR from a Matchmaker Pretransformed Human HeLa library (Clontech) and subcloned into pcDNA3.1. The vectors for the expression of HA-ERp44-WT, C29S, and HA-ERp57 were as described previously (9). DsRed2-ER was purchased from Clontech. ERp44 C29A was generated by site-directed mutagenesis: (forward, 5′-GTA AAT TTT ATG CTG ACT GGG CTC GTT TCA GTC AGA TGT TGC-3′; reverse, GCA ACA TCT GAC TGA AAC GAG CCC AGT CAG CAT AAA AAT TTA C-3′). The ER-targeted redox-sensitive GFP iE variant (ERroGFPiE) was generated from ERroGFPiL (kind gift from Prof. Neil J. Bulleid) by site-directed mutagenesis: (forward, 5′-GGA ATA CAA CTA TAA CTG CGA AAG CAA TGT ATA CAT CAC GGC AG-3′; reverse, 5′-CTG CCG TGA TGT ATA CAT TGC TTT CGC AGT TAT AGT TGT ATT CC-3′).

##### Transfection, Secretion Assay, and Western Blot

Plasmids and siRNAs were transfected using Effectene® (Qiagen) or Lipofectamine RNAiMAX (Invitrogen), respectively, according to the manufacturer's instructions. For secretion assays, cells were incubated in Opti-MEM for an additional 4–6 h. Secreted materials were precipitated with 15% trichloroacetic acid (TCA) or immunoprecipitated with antibodies and then resolved by SDS-PAGE under reducing or nonreducing conditions. For detection of ERroGFPiE, lysates immunoprecipitated with anti-GFP were loaded. Fluorograms or Western blot images were acquired with the ChemiDoc-It imaging system (UVP, Upland, CA) or with the FLA-9000 Starion (Fujifilm Life Science) and quantified with ImageQuant 5.2 as described by Anelli *et al.* (7). Cells were extracted with 1% Nonidet P-40, 150 mm NaCl, 50 mm Tris-HCl (pH 8.0), and 20 mm *N*-ethylmaleimide. The detergent-soluble fractions of cell lysates were analyzed by Western blot.

##### Oligonucleotides

Stealth^TM^ RNA siRNAs were obtained from Invitrogen. The sequences were as follows: siPDI-1, 5′-AAU GGG AGC CAA CUG UUU GCA GUG A-3′; siPDI-2, 5′-AUA AAG UCC AGC AGG UUC UCC UUG G-3′; siERp44-1, 5′-AUA GAG UAU ACC UAU AUU CAC UGG G-3′; siERp44-2, 5′-UUA AUU GCC GAG CUA CUU CAU UCU G-3′; and siEro1α, 5′-GGG CUU UAU CCA AAG UGU UAC CAU U-3′. Medium GC Stealth^TM^ RNAi duplexes were used as negative controls.

##### LC-MS/MS Analysis

Immunoprecipitation was coupled with custom-made direct nano-flow liquid chromatography-tandem mass spectrometry system (Tokyo, Japan). FLAG-tagged Prx4 and mutants thereof were expressed in HEK293 cells and immunoprecipitated with anti-FLAG. Immunoprecipitates were eluted with FLAG peptides and digested with Lys-C endopeptidase (*Achromobacter* protease I). Cleaved fragments were directly analyzed by a direct nano-flow liquid chromatography-tandem mass spectrometry (LC-MS/MS) system as described previously (23). Assays were repeated at least four times.

##### Immunofluorescence

HeLa cells were washed with phosphate-buffered saline (PBS) and fixed with 4% paraformaldehyde for 20 min at room temperature. Cells were permeabilized with 0.2% Triton X-100 in PBS at room temperature for 5 min followed by incubation in 1% normal goat serum and 1% bovine serum albumin for 1 h. Cells were incubated with primary antibodies for 1 h and then with Alexa Fluor-conjugated secondary antibodies (from Invitrogen Molecular Probes) for 1 h, as indicated. Confocal images were obtained using a LSM 700 confocal microscope and analyzed by the Zen 2009 software (Carl Zeiss, Jena, Germany).

##### Preparation of Human Recombinant Prx4, Ero1α, PDI, and ERp44

Recombinant Ero1α and PDI were described previously (17, 24). Prx4 and ERp44 were expressed in *Escherichia coli* BL21 (DE3) cells (Novagen) by induction with 0.3 mm isopropyl-1-thio-β-d-galactopyranoside at 30 °C for 6 h just after the *A*_600_ reached 0.6. Harvested cells were sonicated in 20 mm HEPES (pH 7.5) containing 20 mm imidazole and 150 mm NaCl. The supernatant from cell lysates was loaded onto a HisTrap column (GE Healthcare) equilibrated with cell suspension buffer and eluted with the same buffer containing 0.5 m imidazole. Eluted fractions were loaded onto a HiLoad 16/60 Superdex 200pg isofraction column equilibrated with 20 mm HEPES-NaOH (pH 7.5) containing 150 mm NaCl. Eluted fractions containing oxidoreductases were desalted and loaded onto a Resource Q column (GE Healthcare) equilibrated with 20 mm Tris-HCl (pH 8.0). Fractions were eluted by a linear gradient of NaCl. Purified proteins were concentrated and stored at −80 °C.

##### Surface Plasmon Resonance (SPR) Measurement

SPR analyses were performed as described previously (17, 24). Briefly, association or dissociation rate constants (*k*_on_ or *k*_off_) to immobilized Ero1α (WT) or Prx4 were determined by SPR measurements on a ProteOn XPR36 protein interaction array system (Bio-Rad). Ero1α (WT)/Prx4 were coupled to the GLC sensor chip (Bio-Rad) through amine coupling chemistry. As a control, one channel was coupled with BSA to exclude background binding. Sensorgrams were recorded simultaneously for several concentrations (0.444–36 μm, in a 3-fold dilution series) of purified oxidoreductases at 25 °C for a 2-min association phase followed by a 10-min dissociation phase with 20 mm HEPES-NaOH (pH 7.4 or pH 6.4), 150 mm NaCl, 0.001% Tween, and 2 mm EDTA as running and sample buffer. Sensorgrams were analyzed by nonlinear regression analysis according to a two-state model by the ProteOn Manager version 3.0 software (Bio-Rad). Experiments were replicated at least three times.

##### Statistical Analysis

All data are presented as the means ± S.E. Statistical significance of the difference between groups was evaluated using Student's *t* test. *p* < 0.05 was considered significant. *, *p* < 0.05, **, *p* < 0.01, ***, *p* < 0.001.

##### Homologous Gene Analysis

To gain an evolutionary perspective, we searched and statistically analyzed homologous genes of ER oxidoreductases using the Kyoto Encyclopedia of Genes and Genomes (KEGG) database (25). The National Center for Biotechnology Information (NCBI) database was also searched for analysis of several sequences (www.ncbi.nlm.nih.gov/protein/).

## RESULTS

### 

#### 

##### Interactions of Prx4 and Ero1α with PDI Family Proteins

**FIGURE 1.**
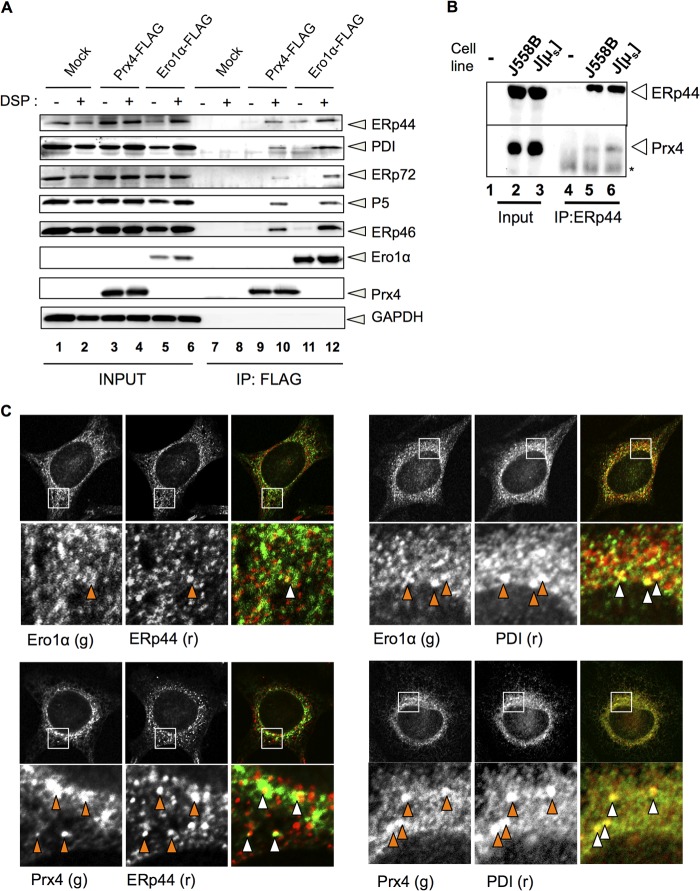
**Prx4 and Ero1α share similar partners and subcellular localizations.** *A*, 24 h after transfection with pcDNA3.1, Prx4-FLAG, or Ero1α-FLAG, 10^6^ HeLa cells were incubated with or without 0.25 μm dithiobis succinimidyl propionate (*DSP*) on ice. Anti-FLAG immunoprecipitates (*IP*) were then eluted by FLAG peptides and analyzed by Western blot with the indicated antibodies. Aliquots of the total Nonidet P-40 lysates from 10^4^ cells (*INPUT*) were loaded to estimate (co)-immunoprecipitation efficiency. *B*, lysates from 10^7^ mouse myeloma J558L cells or their derivative expressing nitrophenol-specific secretory Ig-μ chains (*J[*μ*_s_]*) were immunoprecipitated with anti-ERp44 and analyzed by Western blot with the indicated antibodies. The slightly more abundant Prx4 associated to ERp44 in J[μ_s_] cells may reflect physiological interactions in the presence of an abundant substrate (7). *C*, HeLa cells were fixed by 4% paraformaldehyde and permeabilized by 0.2% Triton X-100. Co-localization of Prx4 or Ero1α with PDI or ERp44 was observed by immunofluorescence using the indicated fluorochrome-conjugated antibodies, as described under “Experimental Procedures.” *g,* green. *r,* red.

The ER oxidases Ero1α and Prx4 have at least two common features; one is their function in oxidative protein folding, and the other is their lack of intrinsic ER retention signals. Surprisingly, the latter feature is 100% conserved among Ero1α orthologs and 94.4% conserved among Prx4 in vertebrates (KEGG database (25)) (supplemental Fig. 1). To identify proteins involved in its subcellular localization, we performed LC-MS/MS analyses of the material co-immunoprecipitated with FLAG-tagged Prx4 and identified ERp44, PDI, ERp72, ERp46, and P5 (supplemental Fig. 2) (see also Ref. 18), yielding a pattern similar to what is reported for Ero1α. To further compare the interactomes of the two enzymes and provide additional specificity controls, we overexpressed Prx4-FLAG or Ero1α-FLAG in HeLa cells and analyzed the immunoprecipitates obtained with or without prior cross-linking with dithiobis succinimidyl propionate. Western blot analyses of the material specifically eluted with FLAG peptides confirmed that both Prx4 and Ero1α interact with ERp44, PDI, ERp72, P5, and ERp46 (Fig. 1*A*). The similar binding patterns are in line with coordinated roles of Prx4 and Ero1α in oxidative protein folding (26).

To confirm that endogenous ERp44 and Prx4 interact in physiological conditions, we analyzed Ig-λ producing J558L murine myeloma cells or a transfectant secreting IgM (J[μ_s_] (27)). Clearly, endogenous Prx4 can be co-immunoprecipitated with ERp44 in Ig-secreting cells (Fig. 1*B*).

Next, we investigated whether Ero1α and Prx4 co-localize with ERp44 or PDI by immunofluorescence (Fig. 1*C*). Although PDI is primarily localized in the ER, endogenous ERp44 recycles between the ER and cis Golgi and accumulates preferentially in the ERGIC (6, 7). Consistent with the results shown in Fig. 1*A*, both Ero1α and Prx4 showed co-localization with PDI and ERp44 in HeLa cells (Fig. 1*C*). Co-localization was stronger with PDI, suggesting that Ero1α and Prx4 were mainly localized in the ER and to a lesser extent in the ERGIC. In many cells, co-staining with ERp44 and PDI was more evident for Prx4 than Ero1α (data not shown), which may reflect the localization of part of Ero1α in mitochondria-associated ER membranes (28, 29).

##### Secretion of Overexpressed Prx4 Is Inhibited by ERp44 and PDI

**FIGURE 2.**
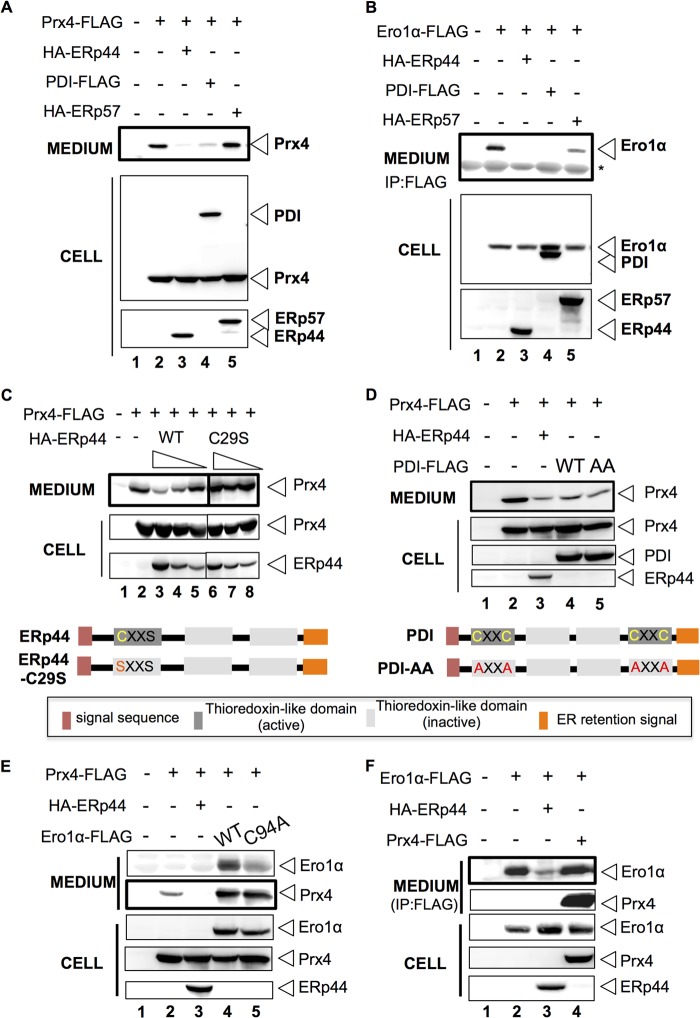
**Dynamic retention of Prx4 by ERp44 and PDI.** *A* and *B*, HeLa cells were co-transfected with Prx4-FLAG (*A*) or Ero1α-FLAG (*B*) and HA-ERp44, PDI-FLAG, or HA-ERp57 as indicated. 24 h after transfection, cells were cultured in FBS-free Opti-MEM medium for 5 h. The spent medium was subsequently precipitated with 15% TCA (*A*) or anti-FLAG antibodies (*B*) and analyzed by Western blot with the indicated antibodies. *C*, Prx4-FLAG was co-expressed in HeLa cells with increasing amounts of HA-ERp44-WT or the C29S mutant (9). 24 h after transfection, cells were handled as described for *panels A* and *B*. When compared with cells overexpressing Prx4-FLAG alone (*lane 2*), Prx4 secretion was inhibited by high levels of ERp44-WT (*lanes 3–5*) but not by ERp44-C29S (*lanes 6–8*). *D*, wild type (PDI-WT-FLAG) or a mutant PDI (PDI-AA-FLAG) in which all four cysteines in the a- and a′-domains had been mutated to alanine were co-expressed with Prx4-FLAG in HeLa cells and handled as above. When compared with cells overexpressing Prx4-FLAG alone (*lane 2*), both PDI-WT and the AA mutant retained Prx4 (*lanes 4* and *5*). *E* and *F*, wild type (Ero1α-FLAG) or an enzymatically inactive variant (Ero1α-C94A-FLAG) was co-expressed with Prx4 in HeLa cells. Clearly, Prx4 secretion was dramatically increased by co-expression of either Ero1α-FLAG or Ero1α-C94A-FLAG. In the experiment shown in *panel F*, Prx4-FLAG was co-expressed with Ero1α-FLAG in HeLa cells. Unlike what observed in *panel E*, retention of Ero1α was not competed by Prx4-FLAG co-expression.

Confirming previous observations (30), overexpressed Prx4 was clearly secreted by HeLa cells (Fig. 2*A*, *lane 2*), implying that saturable mechanisms determine its intracellular retention. Co-expression of ERp44 or PDI, but not of ERp57, restored retention of overexpressed Prx4 (Fig. 2*A*, *lanes 3–5*). These secretory phenotypes were similar for Ero1α (Fig. 2*B*). In the experiment shown, ERp57 partly inhibited secretion of overexpressed Ero1α, albeit much less efficiently than ERp44 or PDI (Fig. 2*B*, *lane 5*) (20). ERp57 cooperates with calnexin and calreticulin to promote glycoprotein folding. The absence of glycosylation sites in Prx4 may explain why co-expressed ERp57 did not affect its secretion. Thus, ERp44 and PDI but not ERp57 can retain overexpressed Prx4.

In thiol-dependent quality control, Cys-29 in the atypical redox-active motif of ERp44 forms mixed disulfides with Ero1 and client proteins such as IgM, adiponectin, or SUMF1/FGE (sulfatase-modifying factor 1/formylglycine-generating enzyme) (5). Clearly, Prx4-FLAG secretion was decreased in a dose-dependent manner by wild type HA-ERp44 (WT) but not by a mutant in which Cys-29 was replaced by a serine (yielding ERp44 C29S, Fig. 2*C*). In contrast, a PDI mutant in which cysteine residues of the two C*XX*C motifs were replaced by alanine residues (PDI-AA) inhibited Prx4 secretion almost as efficiently as wild type molecules (Fig. 2*D*). Thus, the enzymatically active cysteine residues of PDI are not necessary for retention of Prx4.

Because Prx4 shares similar retention mechanisms with Ero1α, the two proteins could compete with each other. Accordingly, secretion of Prx4-FLAG was dramatically increased by Ero1α-FLAG co-expression (Fig. 2*E*, compare *lanes 2* and *4*). Also an enzymatically inactive mutant of Ero1α (Ero1α-C94A) promoted Prx4 secretion (Fig. 2*E*, *lane 5*). Conversely, secretion of Ero1α-FLAG was not increased by co-expression of abundant Prx4-FLAG (Fig. 2*F*, *lanes 2* and *4*).

##### PDI Preferentially Retains Ero1α, whereas ERp44 Equally Retains Ero1α and Prx4

**FIGURE 3.**
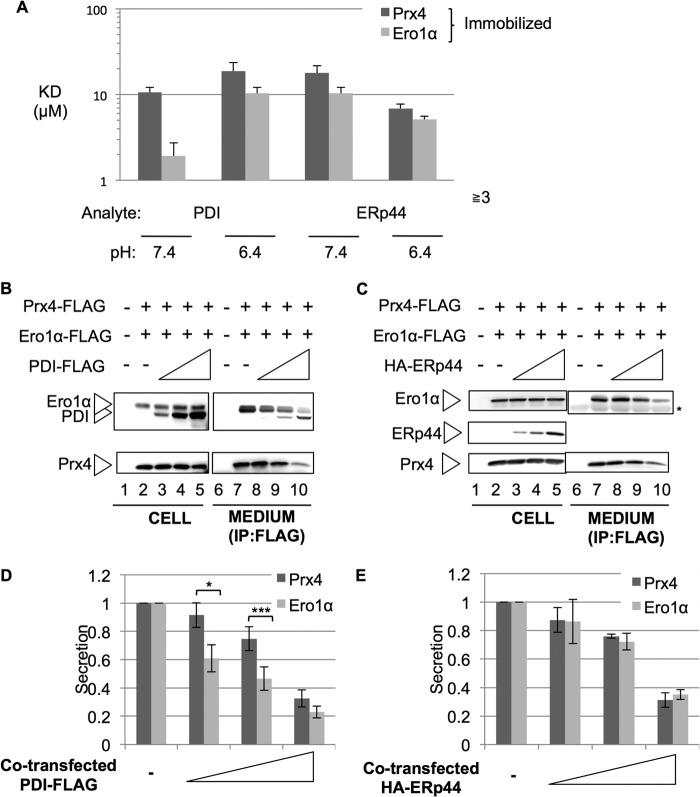
**Ero1α competes with Prx4 for PDI but not for ERp44 binding.** *A*, purified human Ero1α or Prx4 proteins were immobilized on a biosensor chip, and PDI or ERp44 was injected as analyte. The affinity of PDI for Ero1α is about 5.5-fold stronger than Prx4, whereas ERp44 interacts similarly with Ero1α or Prx4. *B–E*, Prx4-FLAG and Ero1α-FLAG were co-expressed with increasing amounts of PDI-FLAG (*B* and *D*) or HA-ERp44 (*C* and *E*) in HeLa cells. 24 h after transfection, cells were cultured in Opti-MEM for 4 h. Aliquots from cell lysates or anti-FLAG immunoprecipitates (*IP*) from the spent medium were analyzed by Western blotting (*D* and *E*) and quantified by densitometry. *n* = 3. *, *p* < 0.05, ***, *p* < 0.001.

The unidirectional competition between Ero1α and Prx4 suggested that the former binds to its retainers more efficiently than the latter. Therefore, we analyzed their binding properties *in vitro* by surface plasmon resonance (SPR) assays and estimated the *k*_on_, *k*_off_, and *K_D_* values. PDI bound Ero1α with ∼5.5-fold stronger affinity than Prx4 at pH 7.4, which is similar to the pH in the ER (1.94 and 10.6 μm, respectively, Fig. 3*A* and supplemental Fig. 3). In contrast, the two enzymes displayed similar affinities for ERp44 at pH 6.4 (5.15 and 6.92 μm, for Ero1α and Prx4, respectively). The affinity of ERp44 to Ero1α and Prx4 was decreased at pH 7.4 in comparison with that at pH 6.4 (Fig. 3*A* and supplemental Fig. 3), suggesting that ERp44 binds Prx4 more effectively at low pH like in the distal ESC stations (10.4 and 17.9 μm for Ero1α and Prx4, respectively) (12). Extrapolating these *in vitro* results to the cellular environment, PDI would preferentially retain Ero1α in the ER.

To challenge this possibility, we co-expressed increasing amounts of PDI-FLAG with constant levels of Ero1α-FLAG and Prx4-FLAG in HeLa cells. 24 h after transfection, culture media and cell lysates were analyzed by Western blot (Fig. 3*B*) and quantified (Fig. 3*D*). Consistent with the *in vitro* results shown in Fig. 3*A*, Ero1α secretion was primarily inhibited by PDI, whereas higher levels of expression of PDI were required to retain Prx4 (Fig. 3, *B* and *D*). In contrast, HA-ERp44 inhibited secretion of Ero1α-FLAG and Prx4-FLAG to similar extents. Collectively, these results indicate that PDI binds and retains Ero1α more efficiently than Prx4.

##### Sequential Interactions of Ero1α and Prx4 with PDI and ERp44 in ESC

**FIGURE 4.**
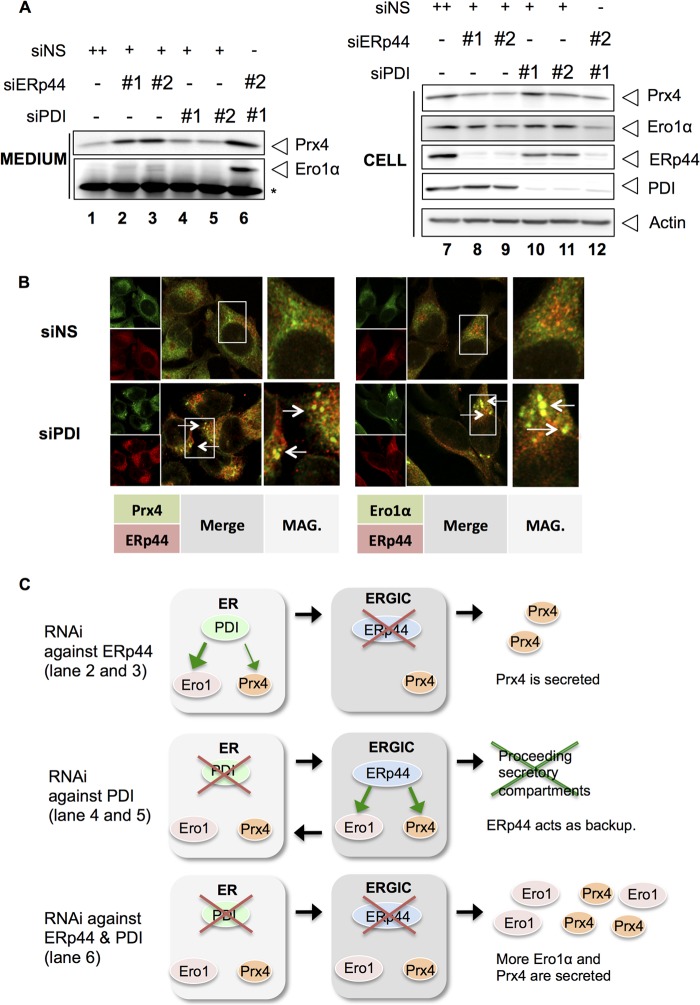
**Silencing ERp44 allows secretion of endogenous Prx4, but not Ero1α.** *A*, secretion of endogenous Prx4 or Ero1α by HeLa cells was analyzed with RNAi for nonspecific (*NS*) ERp44 or PDI (*lanes 1–5*) or both (*lane 6*) by specific siRNAs. 72 h after transfection, cells were cultured in Opti-MEM for 6 h and analyzed as described in the legend for Fig. 2. *B*, immunofluorescence of HeLa cells transfected with nonspecific siRNA (*siNS*) or PDI siRNA (*siPDI*). Endogenous Prx4 or Ero1α was co-stained with endogenous ERp44. In PDI-silenced cells the co-localization of Prx4 or Ero1α with ERp44 was more intense, consistent with a backup role of ERp44. *siERp44*, ERp44 siRNA. *C*, strategy utilized to dissect the retention of Ero1α and Prx4.

**FIGURE 5.**
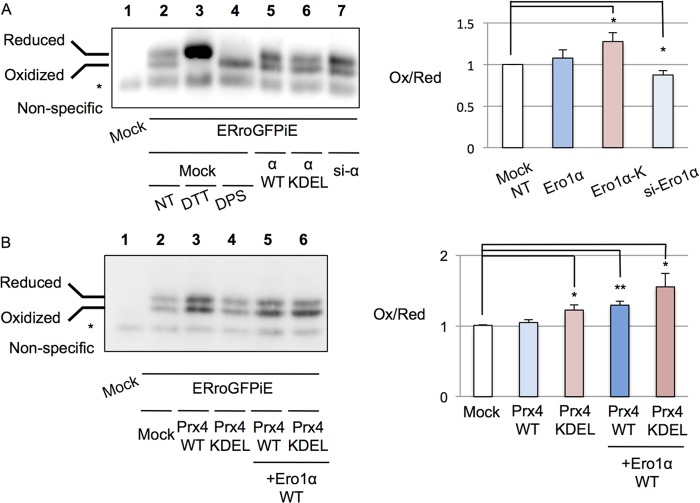
**Increased oxidation of ERroGFPiE upon co-expression of Ero1αKDEL and/or Prx4KDEL.** *A* and *B*, ERroGFPiE is transiently overexpressed in HeLa cells. Reductive or oxidative shift in the ER redox of cells indicated was detected in nonreducing Western blot and quantified. The average ratios of intensity of the oxidized band to the reduced band are depicted in graphs, which are standardized by the ratio of samples of nontreated (*NT*) cells (*lane 2*). *n* = 3. *, *p* < 0.05, **, *p* < 0.01. *DPS*, dipyridyl disulfide; *si-*α, Ero1α siRNA; *si-Ero1*α, Ero1α siRNA; *Ox/Red*, oxidized/reduced.

In view of their different distributions along ESC (7, 8), PDI and ERp44 might exert sequential effects on the localization/retention of Ero1α and Prx4. Therefore, we compared the effects of silencing ERp44, PDI, or both on the secretion of endogenous Prx4 and Ero1α by HeLa cells (Fig. 4*A*). Individual siRNAs for ERp44 or PDI effectively silenced the respective targets (Fig. 4*A*, *lanes 7–12*, *right panel*). Lowering the levels of ERp44 greatly promoted secretion of endogenous Prx4 (Fig. 4*A*, *lanes 1–3*, *upper*), but only marginally affected Ero1α retention (Fig. 4*A*, *lanes 1–3*, *lower*, and Fig. 4*C*, *upper*). Thus, under physiological conditions, PDI seems to retain Ero1α sufficiently. Neither endogenous Prx4 nor Ero1α was released by lowering the levels of PDI alone in HeLa cells (Fig. 4*A*, *lanes 4* and *5*, and Fig. 4*C*, *middle*). Considering that ERp44 is localized downstream with respect to PDI in the ESC, we surmised that ERp44 acted as a backup retention machinery in the absence of PDI (Fig. 4*C*, *middle*). Accordingly, the simultaneous silencing of ERp44 and PDI allowed secretion of both endogenous Ero1α and endogenous Prx4 by HeLa cells (Fig. 4*A*, *lane 6*). Backup mechanism by ERp44 was further confirmed by immunofluorescence of HeLa cells transfected with nonspecific siRNA or specific PDI. Endogenous PDI was efficiently silenced by RNAi (supplemental Fig. 4). As expected, co-localization of ERp44 with Ero1α and Prx4 was increased in PDI-silenced cells (Fig. 5*B*), whereas such a condition did not affect the morphology of the ER or ERGIC (supplemental Fig. 4), suggesting that retention of Ero1α and Prx4 in ESC depends mostly on ERp44 in the absence of PDI. Thus, sequential interactions with PDI and ERp44 underlie the intracellular retention of Prx4 and Ero1α. Ero1α displays higher affinity for PDI, but in its absence, it can be retrieved by ERp44. On the other hand, Prx4 is mainly retained by ERp44 because of its lower affinity for PDI (Fig. 3*A*).

##### Lack of ER Retention Signals in Two ER Oxidases Is Important for ER Redox Homeostasis

In virtually all vertebrates, Ero1α and Prx4 do not harbor ER retention signals (25) (supplemental Fig. 1). As Ero1α and Prx4 play major roles in oxidative protein folding, we surmised that the stepwise retention/localization mechanism of these two ER oxidases in higher eukaryotes may be important for ER redox regulation. To monitor ER redox balance, therefore, we exploited ERroGFPiE. This sensor co-localized with ER-targeted DsRed2 (supplemental Fig. 5). As shown by Birk *et al.* (31), ERroGFPiE can be resolved into two bands under nonreducing conditions corresponding to its reduced (*i.e.* DTT-treated) and oxidized (*i.e.* dipyridyl disulfide-treated) isoforms (Fig. 5*A*, *lanes 2–4*). As indicated by the accumulation of reduced ERroGFPiE and consistent with the notion that Ero1α is a prominent ER oxidase, its knockdown caused hypo-oxidizing condition in the ER (Fig. 5*A*, *lane 7*). Next, we monitored the ER redox state in cells expressing KDEL-extended or wild type Ero1α. Surprisingly, expression of Ero1α-KDEL caused a more oxidizing shift in ERroGFPiE than wild type Ero1α (Fig. 5*A*, *lanes 5* and *6*). Similar results were obtained appending a KDEL motif to Prx4 (Fig. 5*B*). The co-expression of Ero1α with Prx4-KDEL caused a much more dramatic oxidative shift to the redox balance in the ER (Fig. 5*B*, *lane 6*). Taken together, our results strongly suggest that the dynamic, stepwise retention mechanisms of Ero1α and Prx4 are important for fine-tuning the redox status along the ESC.

## DISCUSSION

Our studies have established that two ER oxidases, Ero1α and Prx4, share a noncanonical retention mechanism in the ER. Knockdown of PDI exerted little effect on the secretion of Ero1α and Prx4, whereas knockdown of ERp44 allowed secretion of endogenous Prx4. This observation suggests that Prx4 retention is controlled mainly by ERp44 under physiological conditions. On the other hand, knockdown of both ERp44 and PDI caused marked secretion of Ero1α and Prx4. The different affinity of PDI for Ero1α and Prx4 partially explains why the former was mainly retained by PDI in the ER. After PDI knockdown, the localization of both Ero1α and Prx4 was changed from an ER pattern to a more vesicular pattern containing ERp44. Taken together, these observations strongly suggest that Ero1α and Prx4 are mainly retained by PDI in the proximal ESC. Because of its lower affinity for PDI, some Prx4 continuously reaches the distal ESC stations, from which it is retrieved by ERp44 in a pH-dependent manner, as described for overexpressed Ero1α or IgM subunits (12). In this scenario, ERp44 acts as a backup system. This multistep retention seems conserved throughout evolution; indeed, almost all vertebrates so far reported lack KDEL-like motifs (supplemental Fig. 1).

It is noteworthy that appending KDEL-like motifs to Ero1α or Prx4 caused hyperoxidizing conditions in the ER (Fig. 5). As suitable redox homeostasis is required for efficient as well as accurate oxidative protein folding in the ER (26), our results argue in favor of a physiological role for the dynamic retention of the two ESC oxidases.

An important result emerging from our studies is that ERp44 binds Prx4 more strongly at acidic pH. ERp44 is a unique PDI family member whose conserved CRFS active motif limits its potential function as an oxidoreductase. As a chaperone cycling in ESC, ERp44 preferentially binds its client proteins in the acidic environment of cis Golgi to retrieve them into the ER (12). Its lower affinity at neutral pH likely favors client release in the ER.

Because of their similar interaction patterns, Ero1α and Prx4 largely co-localize; their vicinity may optimize productive folding while limiting H_2_O_2_ production and oxidative stress. However, H_2_O_2_ is not only a foe, but can be utilized as an intra- or intercellular signaling device (32, 33). Therefore, it will be of interest to determine whether the relative levels of Ero1α, Prx4, and their retainer molecules differ between cell types or differentiation states. Besides its key potential role in maintaining redox homeostasis, the dynamic retention mechanism of Ero1α and Prx4 appears to generate a gradient of the two oxidases within the ESC. Considering its possible regulation by pH, such a gradient might have relevant functional consequences. Ero1α has been detected on platelet surface in association with PDI, where it might regulate integrin function (34). Particularly in cells establishing close contacts (*i.e.* immunological or neural synapses), export of redox-active molecules might regulate the intensity and duration of intercellular cross-talks.

The thiol group (-SH) of peroxidatic cysteine is oxidized by H_2_O_2_ to sulfenic acid (-SOH). At higher concentrations, H_2_O_2_ further oxidizes the sulfenic moieties to sulfinic (-SO_2_H) and then sulfonic acid (-SO_3_H). Prx4 can undergo hyperoxidation in the ER lumen (35); however, no sulfiredoxin activity has been detected so far in the secretory compartment. Therefore, sulfinylated or sulfonylated Prx4 is likely degraded or released, perhaps acting as intercellular signals. Prx4 is retained by thiol-dependent mechanisms (Fig. 1*C*), and modifications of the peroxidatic cysteines might lead to secretion. However, Prx4 release was similar in cells overexpressing wild type Ero1α or an enzymatically inactive mutant (Fig. 2*E*), suggesting that Ero1α does not weaken Prx4 retention via H_2_O_2_ production, but likely via competitive binding. However, additional H_2_O_2_ sources may cause Prx4 hyperoxidation and release (15). It should be important and interesting to examine whether and how the interactive retention mode of Ero1α and Prx4 regulates oxidative folding of nascent proteins and whether and how it can adapt to changing physiological requirements.

## Footnotes

5 The abbreviations used are:

ER: endoplasmic reticulum
ERGIC: ER-Golgi intermediate compartment
Ero1: ER oxidoreductin-1
ESC: early secretory compartment
PDI: protein disulfide isomerase
Prx4: peroxiredoxin 4
roGFP: redox-sensitive green fluorescent protein
LC-MS/MS: liquid chromatography-tandem mass spectrometry.
